# Structure-aware annotation of leucine-rich repeat domains

**DOI:** 10.1371/journal.pcbi.1012526

**Published:** 2024-11-05

**Authors:** Boyan Xu, Alois Cerbu, Christopher J. Tralie, Daven Lim, Ksenia Krasileva

**Affiliations:** 1 Center for Computational Biology, University of California Berkeley, Berkeley, California, United States of America; 2 Department of Mathematics, University of California Berkeley, Berkeley, California, United States of America; 3 Department of Mathematics and Computer Science, Ursinus College, Collegeville, Pennsylvania, United States of America; 4 Department of Plant and Microbial Biology, University of California Berkeley, Berkeley, California, United States of America; Koç University, TÜRKIYE

## Abstract

Protein domain annotation is typically done by predictive models such as HMMs trained on sequence motifs. However, sequence-based annotation methods are prone to error, particularly in calling domain boundaries and motifs within them. These methods are limited by a lack of structural information accessible to the model. With the advent of deep learning-based protein structure prediction, existing sequenced-based domain annotation methods can be improved by taking into account the geometry of protein structures. We develop dimensionality reduction methods to annotate repeat units of the Leucine Rich Repeat solenoid domain. The methods are able to correct mistakes made by existing machine learning-based annotation tools and enable the automated detection of hairpin loops and structural anomalies in the solenoid. The methods are applied to 127 predicted structures of LRR-containing intracellular innate immune proteins in the model plant *Arabidopsis thaliana* and validated against a benchmark dataset of 172 manually-annotated LRR domains.

## Author summary

In immune receptors across various organisms, repeating protein structures play a crucial role in recognizing and responding to pathogen threats. These structures resemble the coils of a slinky toy, allowing these receptors to adapt and change over time. One particularly vital but challenging structure to study is the Leucine Rich Repeat (LRR). Traditional methods that rely just on analyzing the sequence of these proteins can miss subtle changes due to rapid evolution. With the introduction of protein structure prediction tools like AlphaFold 2, annotation methods can study the coarser geometric properties of the structure. In this study, we visualize LRR proteins in three dimensions and use a mathematical approach to ‘flatten’ them into two dimensions, so that the coils form circles. We then used a mathematical concept called winding number to determine the number of repeats and where they are in a protein sequence. This process helps reveal their repeating patterns with enhanced clarity. When we applied this method to immune receptors from a model plant organism, we found that our approach could accurately identify coiling patterns. Furthermore, we detected errors made by previous methods and highlighted unique structural variations. Our research offers a fresh perspective on understanding immune receptors, potentially influencing studies on their evolution and function.

## Introduction

Solenoid domains are a class of protein structures defined by a repeating helical arrangement of their backbone chain. These domains are found in a diverse range of proteins and play important roles in a variety of biological processes, including protein-protein interactions, molecular recognition, and scaffolding [18]. The coil shape of solenoid domains arises from a repeating motif of amino acid residues, known as *tandem repeat units*. The specific amino acid sequence and length of the repeating unit can vary between solenoid domains, resulting in differences in the overall structure and function of the domain. The modular nature of solenoid domains allows for the construction of complex structures by combining different domains in a predictable and controlled manner [6].

Leucine-rich repeat (LRR) domains are a type of curved solenoid domain with repeated units of about 20—30 residues long which contain leucine residues in a beta-strand conformation. These domains are found in a wide range of proteins, including cell surface receptors, enzymes, and structural proteins, and are known to play important roles in protein-protein interactions, signal transduction, and immune recognition [12].

Leucine-rich repeats play a critical role in the function of the NOD-like receptor (NLR) family of proteins in the innate immune system of plants and animals [17]. NLRs are intracellular immune receptors that recognize pathogen-derived molecules and activate downstream signaling pathways to initiate an immune response. NLRs are involved in the recognition of a wide range of pathogens, including bacteria, fungi, and viruses. NLRs typically consist of three domains: an N-terminal domain, a central nucleotide-binding domain, and a C-terminal LRR domain. The LRR domain is responsible for recognizing and binding to pathogen-derived molecules, such as effector proteins or pathogen-associated molecular patterns (PAMPs) [8]. In particular, the LRR domains of plant NLRs are highly diverse and can recognize a wide range of pathogen-derived molecules, allowing plants to mount a robust and specific immune response to a broad range of pathogens. Understanding LRR domains in plant NLRs is important for developing strategies to enhance plant immunity and improve crop resistance to pathogens.

The concave surface of the leucine-rich repeat domain is generally responsible for binding to ligands [11]. The amino acid residues on the concave surface of the LRR domain form a specific pattern of hydrophobic, polar, and charged residues that can interact with specific ligands, such as proteins, peptides, carbohydrates, or nucleic acids. The specificity of ligand binding by LRR domains is determined by the overall shape and chemical properties of the concave surface, which can be highly variable between different LRR-containing proteins [9, 10]. Additionally, LRR domains can contain variable regions and insertions that can modify the binding specificity and affinity of the domain. More recently, studies such as [13] have shown that “post-LRR” domains which lie at the C-terminal end of the LRR are required for successful plant immune response. Accurate annotation of these domains and their constituent repeat units is thus essential to understanding the components which govern protein shape and binding specificity.

Existing methods for annotating LRR domains give unreliable and inconsistent results due to irregularities in sequence motifs. Profile hidden Markov models (HMMs) are widely used, e.g. by HMMER [4], to annotate protein domains in genomic sequences, but they are sensitive to the size and diversity of the protein family being analyzed and do not perform accurately for rapidly-evolving, highly-divergent families such as LRR [14]. Profile HMMs are also unable to delineate tandem repeat units.

An existing tool, LRRPredictor [7], uses an ensemble of 8 machine learning classifiers to determine the residues which comprise the basic LRR motif of the form “LxxLxL” (where “L” refers to Leucine or other hydrophobic amino acid, and “x” can be any amino acid). We found that LRRPredictor often makes mistakes, particularly in identifying divergent motifs near the C- and N-terminal boundaries of the LRR. Because LRRPredictor, like an HMM, is trained on a specific set of LRR sequences taken from Protein Data Bank [20] (PDB), it incorrectly annotates LRR sequences which diverge from its training set.

With AlphaFold 2 [3], a deep-learning-based model, reliable protein structure prediction has become readily available, enabling domain annotation methods with direct access to geometric data from the protein. We leverage this geometric information to annotate essential features of the LRR domain: start/end position, post-LRR detection, repeat unit delineation, and structural irregularities.

From the perspective of differential geometry, a coiling curve in 3D space is characterized by a linearly increasing winding number around a core curve. We therefore detect the coiling LRR region, as the loci where the winding number is sufficiently close to a line of a fixed slope; the post-LRR domain is then decided as C-terminal sequence downstream from the point at which steady winding terminates. The methods section below describes our procedure for computing the winding number across the length of the protein. In contrast to HMM-based or other data driven techniques, our method is completely unsupervised and driven by simple mathematical methods.

## Methods

### Datasets used in this study

161 NLR protein sequences, i.e. *NLRome*, were obtained from the reference proteome of *A. thaliana* Col-0 TAIR10 as described previously using hmmsearch [32] and the extended NB-ARC Hidden Markov Model [2]. Of these 161 NLRs, 127 had AlphaFold-predicted structures available on AlphaFoldDB [3, 30]. The training dataset used for LRRpredictor, which contained manual annotations of LRR motif positions, was downloaded from supplemental data of [7]. We ran AlphaFold 2 prediction on a supercomputer cluster with default parameters and selected the best-scoring model for further analysis. We have included the protein amino acid sequences and corresponding pdb files in the GitHub repository where we host all the code used in this study.

### Outline of methods

**Fig 1:**
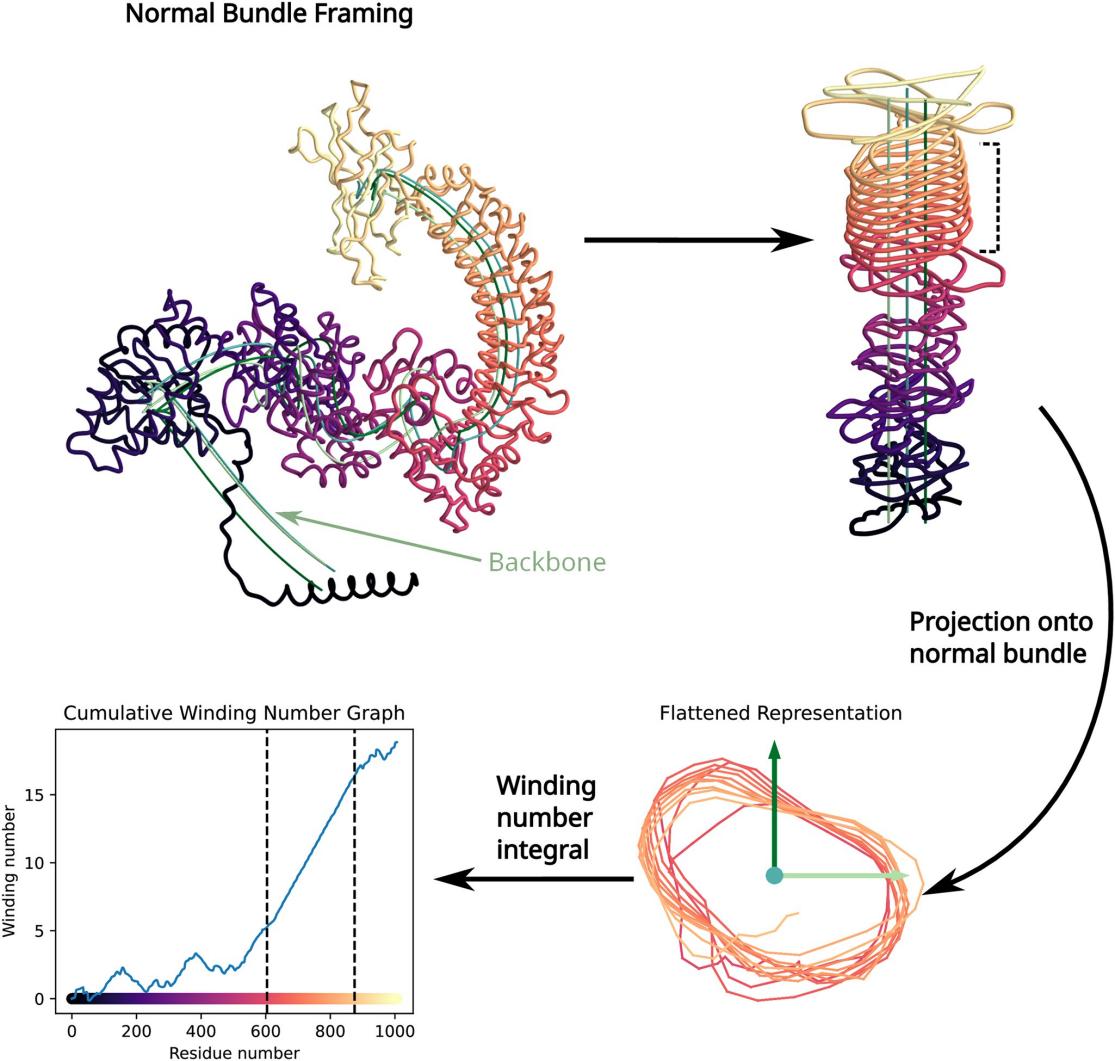
Embedding of protein backbone curve into normal bundle followed by projection onto an orthonormal frame yields a 2D curve containing a flattened slinky shown in lower right. The cumulative winding number, computed using the classic formula from calculus, is computed from the projection. Sloped linear segments of the winding number curve indicate coiling. Protein shown is *A. thaliana* NLR with TAIR [1] ID AT3G44400.2.

**Fig 2:**
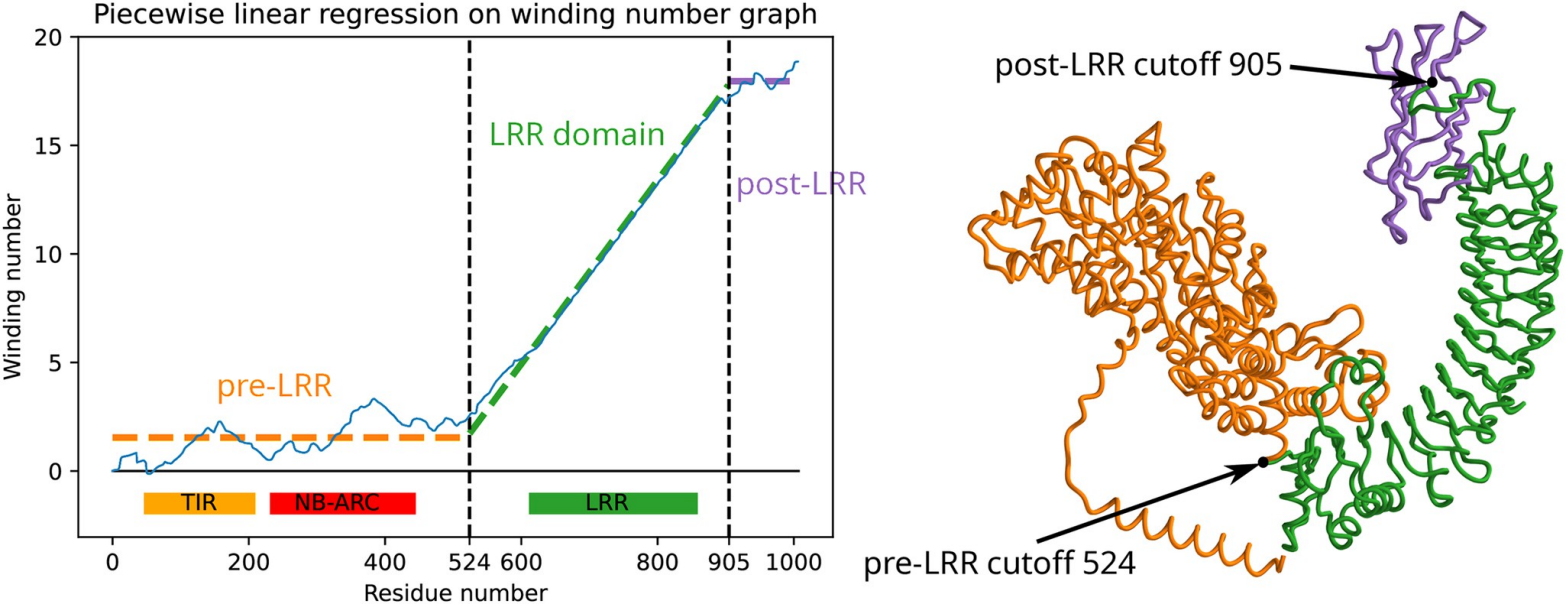
A discontinuous clipped ReLU function is regressed on the graph of the winding number function for *A. thaliana* NLR with TAIR [1] ID AT3G44400.2. The breakpoints of the regression yields the start and end positions of the LRR domain, highlighted in green. InterPro [19] domain annotations are shown below regression plot.

Our treatment of protein structures follows the outline below. Fig 1 shows the results of steps 1–4, while Fig 2 shows the results of steps 5–6.

1. ***Obtaining the backbone***. Given the space curve *γ*(*t*) representing the positions of the *α*-carbons, obtain a smoothed backbone curve *γ*_*σ*_(*t*) by convolving *γ* with a Gaussian.
2. ***Parallel transport & framing***. Parallel-transport a frame along the backbone to produce, at each position *t*, an orthonormal basis for the plane normal to $\gamma_{\sigma }^{\prime }\left(t\right)$. This yields a two-dimensional coordinate system *A*(*t*) for each *t*.
3. ***The flattened representation***. For each *t*, compute the coordinates of *γ*(*t*) − *γ*_*σ*_(*t*) according to *A*(*t*). This produces a two-dimensional “flattened” curve *φ*(*t*) representing the position of *γ* relative to its backbone.
4. ***Cumulative winding number***. Compute the cumulative winding number *W*_*φ*_(*t*) of *φ* about the origin.
5. ***Secant line statistics; median slope***. Compute the median slope of secant lines to *W*_*φ*_ to infer the number *m* of residue positions per helical repeat unit in the LRR domain.
6. ***Piecewise-linear regression & gradient descent***. By gradient descent on an appropriate loss function, find a piecewise-linear regression of *W*_*φ*_ with slopes alternating between zero and *m*. Regions of the regression with slope *m* correspond to solenoidal regions of the protein structure.

### Obtaining the backbone

Let *γ*(*t*), *t* ∈ {0, …, *n*} be a discrete space curve representing the positions of the *α*-carbons in a protein structure. This curve can be represented as three scalar functions of *t*: *γ*(*t*) = (*γ*_*x*_(*t*), *γ*_*y*_(*t*), *γ*_*z*_(*t*)). Let *g*_*σ*_ be the mean-zero Gaussian distribution with standard deviation *σ*:

$$\begin{array}{c} g_{\sigma }\left(t\right)=\frac{1}{\sigma \sqrt{2\pi }}e^{-t^{2}/\left(2\sigma^{2}\right)}. \end{array}$$ (1)

We define the “backbone” to the structure

$$\begin{array}{c} \gamma_{\sigma }\left(t\right)≔(\left(g_{\sigma }⋆\gamma_{x}\right)\left(t\right),\left(g_{\sigma }⋆\gamma_{y}\right)\left(t\right),\left(g_{\sigma }⋆\gamma_{z}\right)\left(t\right)), \end{array}$$ (2)

where ⋆ is the convolution, defined (*p* ⋆ *q*)(*t*) ≔ ∑_*s*_ *p*(*t*)*q*(*t* − *s*), where the sum is over all sensible indices *s*. Throughout in our computations, we set *σ* = 20.

### Parallel transport & framing

First, we compute the tangent vector $\gamma_{\sigma }^{\prime }\left(t\right)$ to the backbone by convolving *γ* with the derivative of a Gaussian, i.e. with

$$\begin{array}{c} g_{\sigma }^{\prime }\left(t\right)=-\frac{t}{\sigma^{3}\sqrt{2\pi }}e^{-t^{2}/\left(2\sigma^{2}\right)}. \end{array}$$ (3)

This is a standard technique [31] for defining derivatives of discrete data, since convolution associates with differentiation as (*d*/*dt*)(*p* ⋆ *q*) = ((*dp*/*dt*) ⋆ *q*) = (*p* ⋆ (*dq*/*dt*)). In order to measure the winding of *γ* around its backbone *γ*_*σ*_, we need a consistent representation of the position of *γ* relative to *γ*_*σ*_; in effect, we need to “straighten” the backbone and carry *γ* along for the ride.

Now that we have $\gamma_{\sigma }^{\prime }\left(t\right)=\left(g_{\sigma }^{\prime }⋆\gamma \right)\left(t\right)$, we will produce a sequence of orthonormal bases for the planes orthogonal to $\gamma_{\sigma }^{\prime }\left(t\right)$ at each residue *t*. Our method starts with a frame at *t* = 0 and parallel-transports it along the backbone as follows:

1. Given $\gamma_{\sigma }^{\prime }\left(0\right)$, the initial tangent to the backbone, let *A*(0) be any 3 × 2 real matrix with orthonormal columns such that $A{\left(0\right)}^{T}\gamma_{\sigma }^{\prime }\left(0\right)=0$ (i.e., the columns of *A*(0) complete $\gamma_{\sigma }^{\prime }\left(0\right)$ to an orthonormal basis for $R^{3}$).
2. Given *A*(*t* − 1), let *B*(*t*) be the matrix whose columns are orthogonal projections of the columns of *A*(*t* − 1) onto the complement of $\gamma_{\sigma }^{\prime }\left(t\right)$. Symbolically,
  $$\begin{array}{c} B\left(t\right)=A\left(t-1\right)-\frac{1}{{\left\|\gamma_{\sigma }^{\prime }\left(t\right)\right\|}^{2}}\gamma_{\sigma }^{\prime }{\left(t\right)}^{T}A\left(t\right)\gamma_{\sigma }^{\prime }\left(t\right). \end{array}$$ (4)
  The columns of *B*(*t*) are likely not orthonormal.
3. Let *A*(*t*) be the 3 × 2 matrix with orthonormal columns that is closest (in the Frobenius norm) to *B*(*t*). Numerically, *A*(*t*) is found by computing the SVD of *B*(*t*) and replacing its singular values with 1’s (the standard solution to the “Orthogonal Procrustes Problem” [15, 16]). Note that the columns of *A*(*t*) span the same subspace as those of *B*(*t*), so *A*(*t*) has columns guaranteed orthogonal to $\gamma_{\sigma }^{\prime }\left(t\right)$.
4. Repeat steps 2 and 3 for *t* = 1, …, *n*.

### The flattened representation

The flattened representation is now a plane curve *φ*(*t*) = *A*(*t*)^*T*^(*γ*(*t*) − *γ*_*σ*_(*t*)). It can be thought of as *γ* from the perspective of an observer traveling along the backbone *γ*_*σ*_ and oriented according to the frames *A*(*t*).

### Cumulative winding number

For a continuous-time plane curve *z*(*t*) = (*x*(*t*), *y*(*t*)) with polar representation (*r*(*t*) cos(*θ*(*t*)), *r*(*t*) sin(*θ*(*t*)), the winding number is defined

$$\begin{array}{c} W_{z}\left(t\right)=\frac{1}{2\pi }\int_{0}^{t}\theta^{\prime }\left(s\right)ds=\frac{1}{2\pi }\int_{0}^{t}\frac{x\left(s\right)y^{\prime }\left(s\right)-y\left(s\right)x^{\prime }\left(s\right)}{x{\left(s\right)}^{2}+y{\left(s\right)}^{2}}ds. \end{array}$$ (5)

This quantity tracks the total number of rotations accumulated by a ray pointing at *z*(*s*), as *s* moves in the interval [0, *t*].

In our case, given the discrete plane curve *φ*(*t*) = (*x*(*t*), *y*(*t*)), we define a discrete version of the cumulative winding number by

$$\begin{array}{c} W_{\varphi }\left(t\right)=\frac{1}{2\pi }\sum_{s=1}^{t}\;\text{arctan}\;(\frac{y(s)x(s-1)-x(s)y(s-1)}{x(s)x(s-1)+y(s)y(s-1)}). \end{array}$$ (6)

The summand accumulates the angle between rays to consecutive points *φ*(*s* − 1) and *φ*(*s*) along the discrete curve. Fig 1 provides a graphical example of the backbone, parallel-transported normal bundle, flattened representation, and cumulative winding number plot.

### Secant line statistics; median slope

To make piecewise-linear regression tractable, we remove slope as an optimization parameter, and instead infer it from the statistics of secant lines to *W*_*φ*_. First we choose parameters 0 < *d* < *D* (in the median_slope method, these are small = 100 and big = 250, respectively). We will consider only secant lines with endpoints *a*, *b* where *d* ≤ *b* − *a* ≤ *D*. Associated to such a secant line is a slope *m*_*a*,*b*_ = (*W*_*φ*_(*b*) − *W*_*φ*_(*a*))/(*b* − *a*), and a score *S*_*a*,*b*_ computed as follows. First define

$$\begin{array}{c} R_{a,b}=\sum_{t=a}^{b}{\left[(W_{\varphi }\left(t\right)-m_{a,b}t)-\frac{1}{b-a+1}\sum_{s=a}^{b}(W_{\varphi }\left(s\right)-m_{a,b}s)\right]}^{2}. \end{array}$$ (7)

In other words, *R*_*a*,*b*_ measures the total squared deviation of (*W*_*φ*_(*t*) − *m*_*a*,*b*_*t*) away from its mean; we have *R*_*a*,*b*_ = 0 if and only if *W*_*φ*_ coincides with its secant line on *t* ∈ [*a*, *b*]. Now let the score *S*_*a*,*b*_ = (*b* − *a*)/(1 + *R*_*a*,*b*_), rewarding long secant lines and penalizing deviations from linear behavior.

The “median slope” is chosen by a voting process. First we determine the minimum and maximum slopes, call them *m* and *M*. We create $\sqrt{N}$ score bins, where *N* is the number of secant lines, i.e. the number of pairs (*a*, *b*) with 0 ≤ *a*, *b* ≤ *n* and *d* ≤ *b* − *a* ≤ *D*. For each secant line with endpoints *a*, *b*, its score *S*_*a*,*b*_ accumulates in the bin with index ⌊(*m*_*a*,*b*_ − *m*)/(*M* − *m*)⌋. After this procedure, the slope returned is $m+(i/\sqrt{N})\left(M-m\right)$, where *i* is the index of the bin with largest score. We use this slope in subsequent regression tasks.

Our “median slope” computation is conceptually similar to the Hough transform [33], a computer vision method for detecting segments in images via a voting process across a parametrized space of lines in the plane.

### Piecewise-linear regression & gradient descent

The “median slope” *m* associated to the winding *W*_*φ*_ approximates the reciprocal of residues per repeat unit in the LRR domain—as residue position *t* changes by *m*, winding number increases by 1, i.e. *φ* completes one revolution around the origin. To annotate the domain in which *W*_*φ*_ exhibits this linear, slope-*m* behavior, we fit a piecewise-linear, discontinuous function which is constant in the pre-LRR region, slope-*m* in the LRR domain, and constant in the post-LRR region. More precisely, associated to a choice of breakpoints (0 = *a*_0_ < *a*_1_ < ⋯ < *a*_*k*_ = *n*) is a regression function that is constant on [*a*_0_, *a*_1_), slope-*m* on [*a*_1_, *a*_2_), constant on [*a*_2_, *a*_3_), and so on. Most of the cumulative winding number plots were well-approximated with *k* = 2 (two breakpoints); we discuss larger *k* below.

We define a loss function, similar in spirit to 7, as follows. First, for a function *f*(*t*) and endpoints *a* < *b*, define *V*_*a*,*b*_(*f*) to be the total squared deviation of *f* from its mean on [*a*, *b*):

$$\begin{array}{c} V_{a,b}\left(f\left(t\right)\right)=\sum_{t=a}^{b-1}{\left(f\left(t\right)-\frac{1}{b-a}\sum_{s=a}^{b-1}\;f\left(s\right)\right)}^{2}. \end{array}$$ (8)

Choose constants *C*, *D*, and define the loss associated to the partition (*a*_0_, …, *a*_*k*_):

$$\begin{array}{c} L\left(a_{0},\ldots ,a_{k}\right)=\sum_{j=0}^{k/2}(C\;V_{a_{2j},a_{2j+1}}(W_{\varphi }\left(t\right))+D\;V_{a_{2j+1},a_{2j+2}}(W_{\varphi }\left(t\right)-mt)). \end{array}$$ (9)

The loss is a weighted measurement of the total squared deviation between *W*_*φ*_ and the regression function we are fitting, with the weights *C* and *D* determining how harshly we penalize deviations from linearity (slope-*m* behavior) in the LRR region. In our code, we found that *C* = 1 and *D* = 1.5 worked well. Our optimization problem now becomes: find (*a*_0_, …, *a*_*k*_) minimizing *L*(*a*_0_, …, *a*_*k*_).

We solve the optimization problem by gradient descent on *L*: we form a finite-difference gradient ∇*L* whose *j*^th^ entry is

$$\begin{array}{c} {\left(\nabla L\right)}_{j}=(L\left(a_{0},\ldots ,a_{j}+1,\ldots ,a_{k}\right)-L\left(a_{0},\ldots ,a_{j},\ldots ,a_{k}\right)), \end{array}$$ (10)

choose a learning rate *ϵ* > 0, increment the vector of breakpoints by −*ϵ*∇*L*, and iterate.

### Refinements and alternatives

#### Loss histograms & four-breakpoint regressions

**Fig 3:**
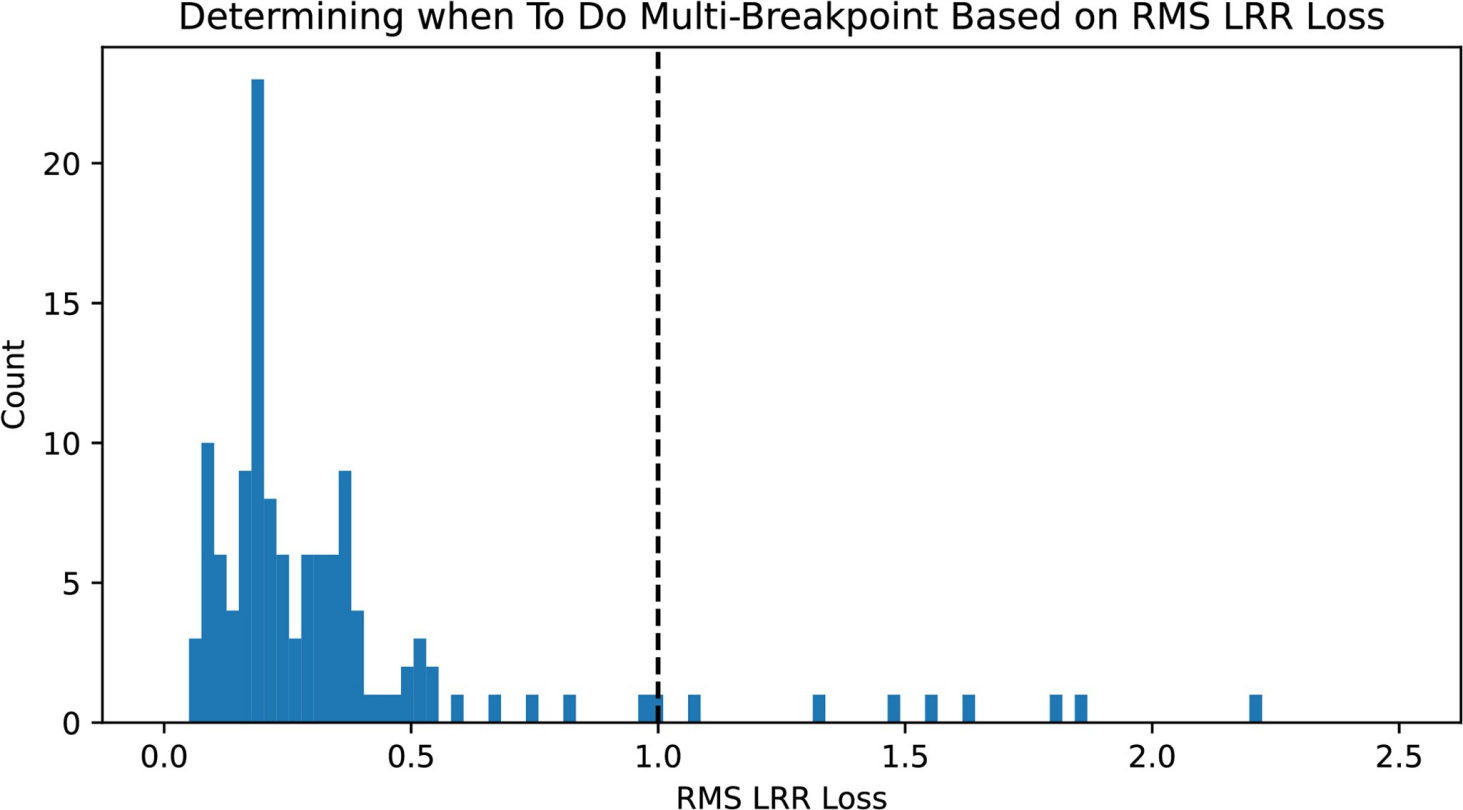
We determine when to redo the regression using 4 breakpoints by examining the RMS of the LRR component of the loss. This term is above our threshold of 1 for 9/127 of the proteins in *A. thaliana*.

A small number (ten out of 127) of proteins in our dataset contained hairpin loops or other localized deviations from solenoidal geometry in the LRR region, and regressions with *k* = 2 breakpoints were not satisfactory. We found the standard deviation of the difference between *W*_*φ*_ and the regressing function inside the LRR region, i.e. $V_{a_{1},a_{2}}/\left(a_{2}-a_{1}\right)$, is high in such cases. Fig 3 shows the distribution of these values. We repeat the regression with four breakpoints, instead of two, to deal with these edge cases.

**Fig 4:**
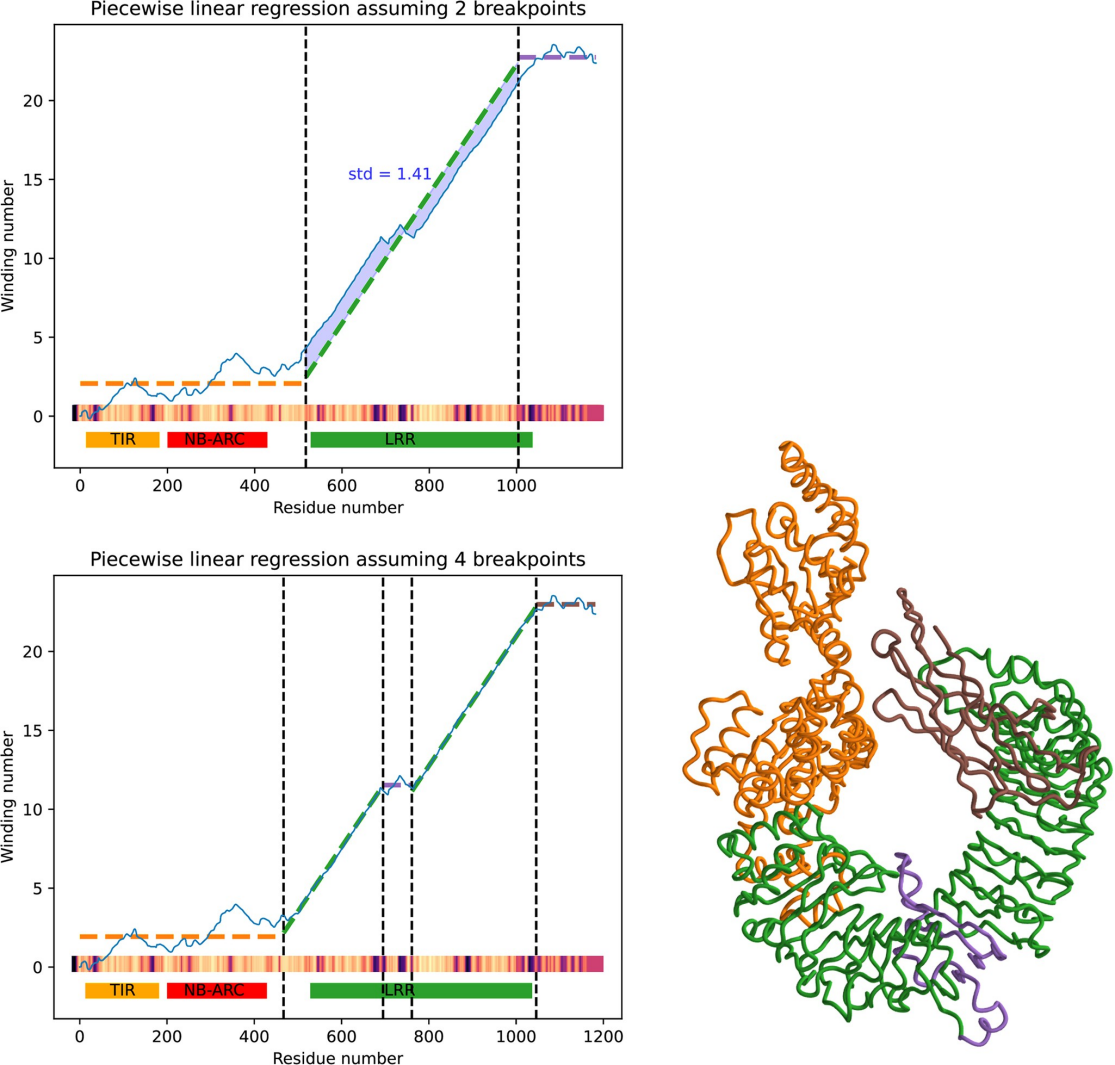
Four breakpoint piecewise linear regression enables detection of a non-coiling structure (highlighted in purple at right) which deviates from the usual coiling in the LRR domain. Below regression plot, a heat map shows the pLDDT (predicted local distance difference test), a per-residue confidence measure given by AlphaFold 2 which is elevated in the non-coiling region. Bottom of plot shows HMM-based InterPro domain annotations which fail to detect non-coiling region within LRR domain. TAIR ID is AT1G72840.2.

Fig 4 shows the result of fitting a regressing function with four, instead of two breakpoints.

#### Laplacian circular coordinates

In the previous sections, we used piecewise linear regression on the cumulative winding number to isolate the LRR domain. In the process, we estimated the winding number, which can also give us *instantaneous phase*, or the angle along each loop, on the LRR domain sequence. In this section, we briefly describe another technique based on graph theory for estimating instantaneous phase of LRR regions, which we evaluate alongside the parallel transport method in Section 7.2.

Before setting up the graph, we perform some preprocessing to make LRR solenoid region as circular as possible. First, we nullify some of the torsion by once again computing the tangent vectors on the LRR solenoid. This time, however, we set *σ* = 1, and convolve *γ*(*t*) with $g_{1}^{\prime }\left(t\right)$ instead of $g_{20}^{\prime }\left(t\right)$ to preserve the loop structures. To further accentuate periodic features, we perform a multivariate sliding window embedding [5] of window size 24 (roughly the length of the LRR period) with delay time 1 on each component of the tangent vector field. The formula for such a sliding window embedding of some sequence *f*[*t*] is

$$\begin{array}{c} {\text{SW}}_{1}^{24}\;f\left[t\right]≔[\begin{array}{c} \gamma [t]\\ \gamma [t+1]\\ \gamma [t+2]\\ \vdots \\ \gamma [t+24] \end{array}]\in R^{24+1} \end{array}$$ (11)

We concatenate together ${\text{SW}}_{1}^{24}\;\gamma_{i\sigma }^{\prime }$ for each of the three components of the tangent vector $\gamma_{i\sigma }^{\prime }$, resulting in a sequence in 75-dimensional Euclidean space. We then construct a 50-mutual-nearest-neighbors graph on the sliding window embedding.

**Fig 5:**
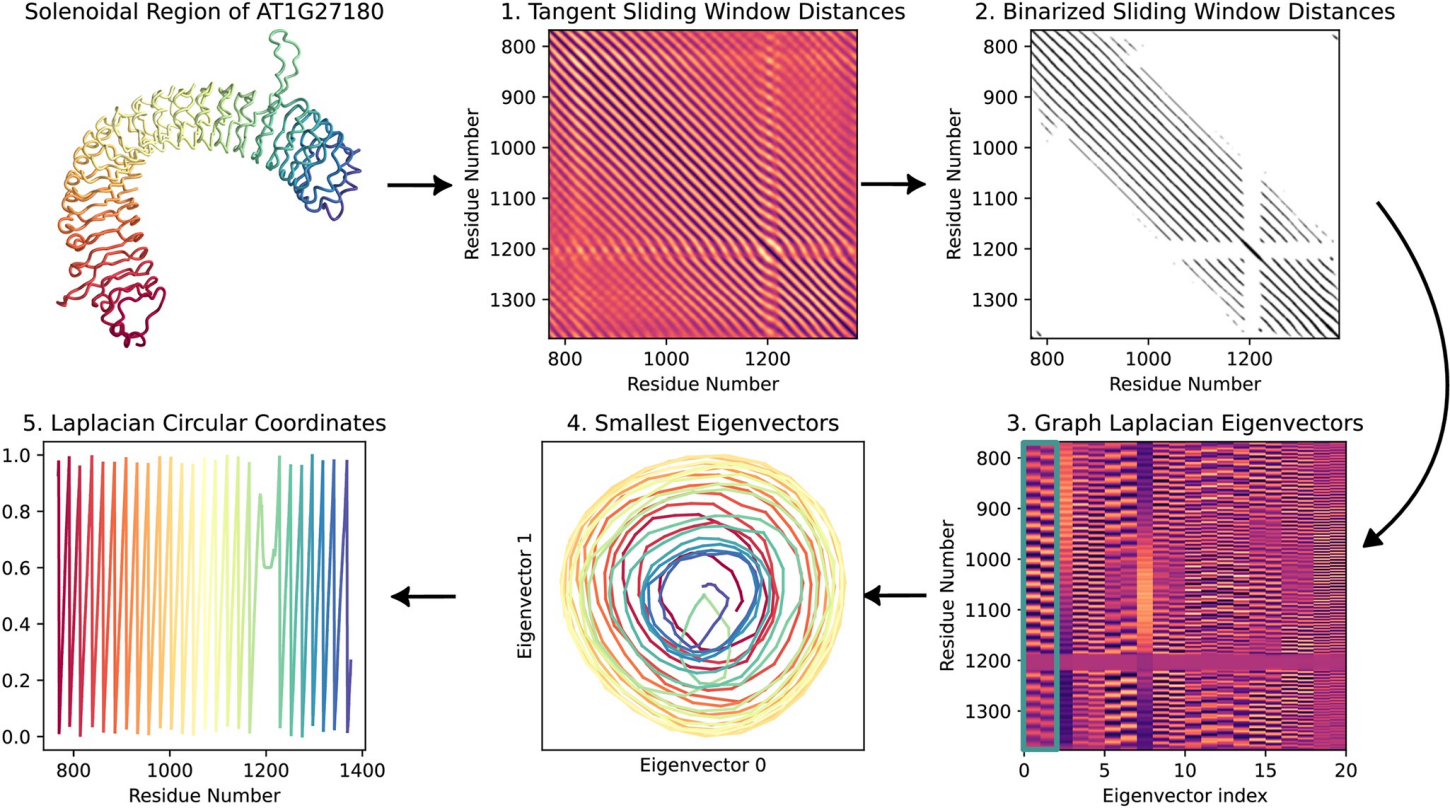
Graph Laplacian eigenvectors of mutual nearest neighbor graph on LRR solenoid curve tangent vectors. LRRPredictor residues are shown as blue horizontal lines on eigenmatrix plot. The 0^th^ and 1^st^ eigenvectors have period matching the expected period of the solenoid as determined by LRRPredictor. Leading eigenvectors of graph Laplacian are periodic and are *π*/4-phase shifted, thereby yielding projections of LRR coil onto a winding around a circle in a 2D-plane. Phase estimation using the formula $\theta ={\text{tan}}^{-1}\;\left(\frac{y}{x}\right)$ of LRR coil at bottom taking values between −*π* and *π*.

From the mutual-NN graph we compute leading eigenvectors of the unweighted graph Laplacian [23]. An example is shown in Fig 5. Intuitively, the graph Laplacian is a generalization of a discrete second derivative operator to graphs. For the same reason that sines and cosines are eigenfunctions of the second derivative operator with associated eigenvalues proportional to the frequency, eigenvectors of the graph Laplacian on a graph of a circle are sine-cosine pairs, up to a phase, that go through an integer number of cycles over one revolution of the circle, and lower frequency pairs have smaller eigenvalues [24]. We expect a near circular graph in the mutual-NN graph in the periodic LRR region, and the Laplacian eigenvectors are known to degrade gracefully in the presence of imperfections. Therefore, we expect the two eigenvectors with the smallest eigenvalue to be approximately periodic and *π*/4-phase shifted. If we use the two entries of these eigenvectors as *x*- and *y*-coordinates, respectively, we obtain a projection of the LRR coil onto a circle winding in the plane. Our phase estimation *θ* along the LRR coil is simply obtained as $\theta ={\text{tan}}^{-1}\;\left(\frac{y}{x}\right)$, as shown in Fig 5 below.

We note that a similar phase-estimation scheme with the graph Laplacian of mutual nearest neighbors has been used to order photographs along a loop [25] and to parameterize periodic videos [5]. Furthermore, a spiritually similar but more computationally intensive topological phase estimation based on cohomology [28, 29] has been used to recognize patterns in motion capture data [26] and to detect head orientation from neural data [27].

## Results

### Cumulative winding number reveals errors made by ML-based LRR repeat unit delineator

We ran the LRR annotation tool LRRPredictor [7] on the 127 NLRs from *A. thaliana* to obtain predicted locations of the LRR motif “LxxLxL.” Let *R*_1_, …, *R*_*ℓ*_ denote the starting residues for the LRR motifs predicted by LRRPredictor. The analogous measurement in our model is to record the residues at which our cumulative winding number *W*_*φ*_ crosses integers.

To compare the two prediction schemes, we evaluate our cumulative winding number at the residues returned by LRRPredictor. That is, we form the list of numbers (*W*_*φ*_(*R*_1_), …, *W*_*φ*_(*R*_*ℓ*_)). If the models are in agreement, the running difference (*W*_*φ*_(*R*_2_) − *W*_*φ*_(*R*_1_), …, *W*_*φ*_(*R*_*ℓ*_) − *W*_*φ*_(*R*_*ℓ*−1_) should equal the all-ones vector (1, …, 1) (that is, the structure should wind exactly once around the core between residues *R*_*j*−1_ and *R*_*j*_). The “discrepancy”

$$\begin{array}{c} D\left(R_{1},\ldots ,R_{\ell }\right)≔\sqrt{\sum_{j=2}^{\ell }{\left(W_{\varphi }\left(R_{j}\right)-W_{\varphi }\left(R_{j-1}\right)-1\right)}^{2}} \end{array}$$ (12)

quantifies the extent to which this is not the case. A number of LRRPredictor outputs contained false predictions in which consecutive motif start sites *R*_*j*_ and *R*_*j*−1_ appear close together—often only a couple residues apart. Such duplicate predictions result in a high discrepancy *D*(*R*_1_, …, *R*_*ℓ*_) because the difference *W*_*φ*_(*R*_*j*_) − *W*_*φ*_(*R*_*j*−1_) as computed in formula (12) above is close to 0.

To test the validity of our winding number computation, we ran the discrepancy computation on the LRRPredictor outputs on the 127 *A. thaliana* reference proteome NLRs as well as AlphaFold 2 structures for the training dataset for LRRpredictor, a manually-annotated “ground truth” dataset of LRR motifs on 172 experimentally-derived LRR structures taken from Protein Data Bank. These PDB protein structures were derived from a diverse set of organisms comprising bacteria, fungi, plants, and animals.

**Fig 6:**
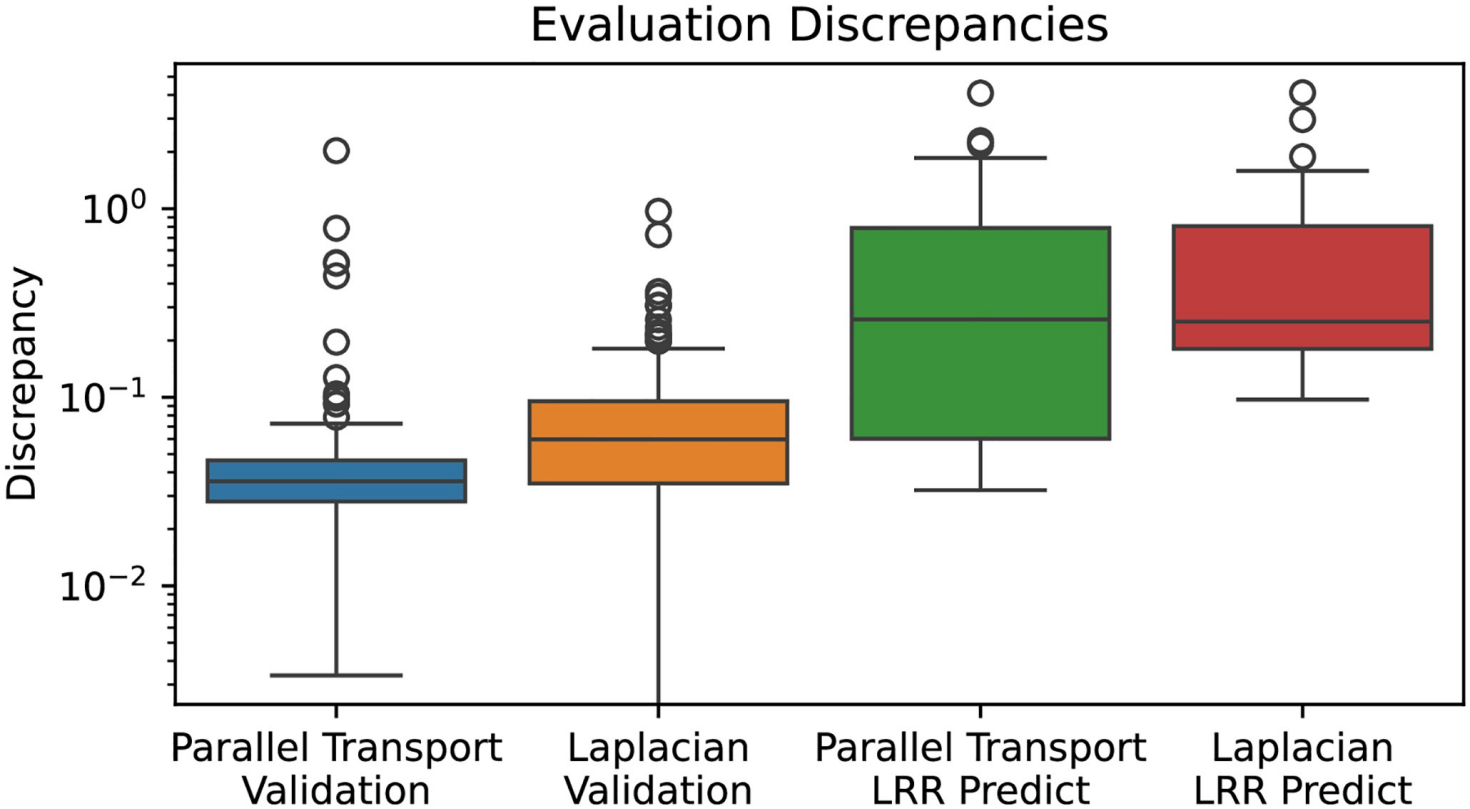
Discrepancies for LRRpredictor outputs on 127 *A. thaliana* (green and red) NLRs are higher than those for manually-annotated LRR repeat units used as the training set for the LRRpredictor model (blue, orange). Thus, the cumulative winding number computation faithfully recapitulates the periodicity of the LRR coil.

**Fig 7:**
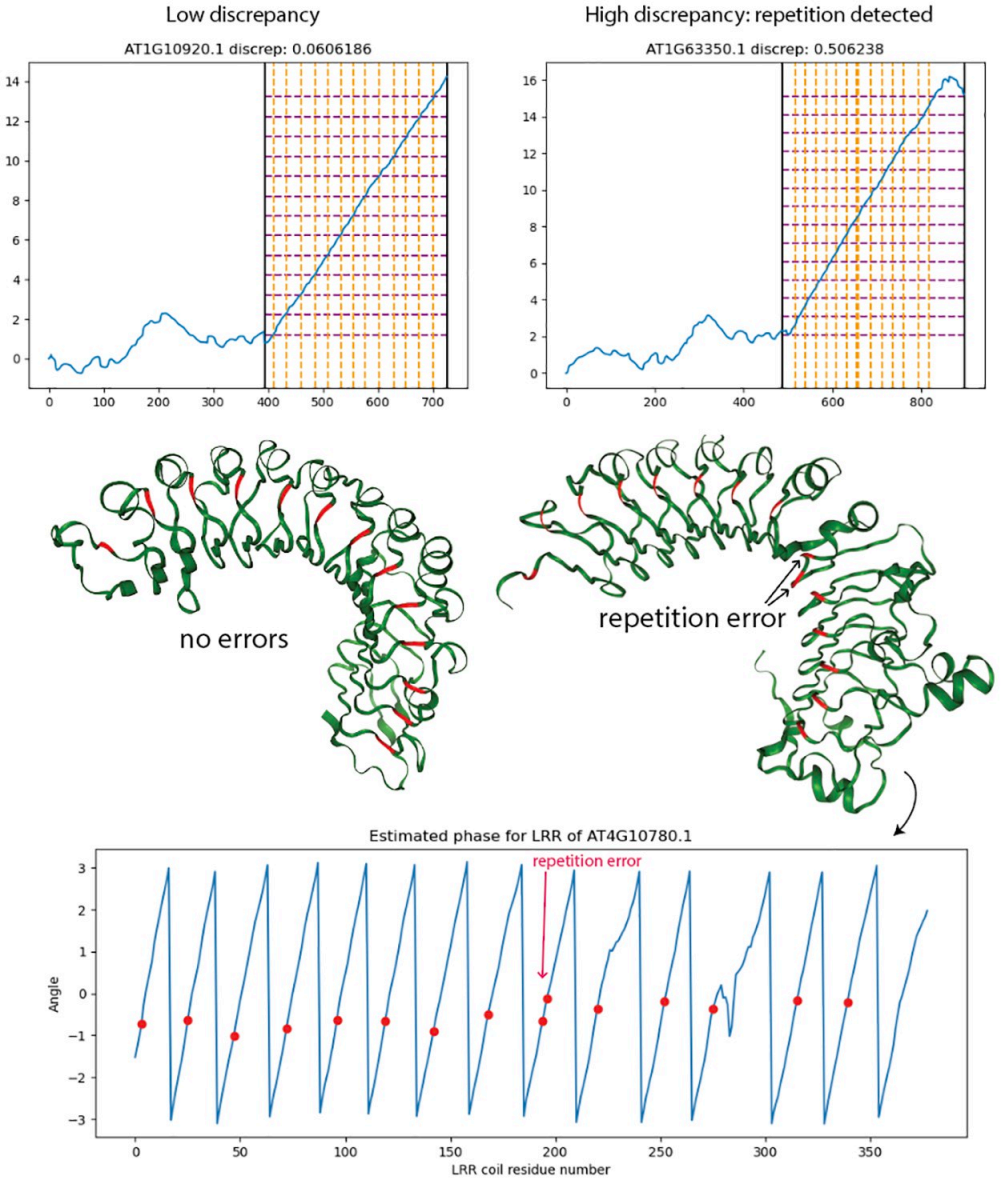
LRRPredictor discrepancy computation reveals proteins with erroneously repeated predictions. NLRs with high-discrepancy LRRPredictor outputs tend to carry repetition errors or missing motif annotations. Orange vertical lines overlaid on winding number plot depict LRRPredictor residues, while purple horizontal lines depict the integer-spaced grid which best approximates the winding number graph evaluated at LRRPredictor residues. A repetition error can be seen in the grid representation as a doubled orange line around residue 685. At bottom, LRRPredictor residues are mapped onto graph Laplacian eigenvector phase estimation, revealing an pair of duplicates with adjacent phase.

We found consistently low discrepancy values for the ground truth set with mean 0.127. By comparison, *A. thaliana* NLRome discrepancy values were generally low with mean 0.373, but exhibited higher values in cases where LRRpredictor made mistakes. Fig 6 below shows a pair of overlaid histograms comparing discrepancy values for both the NLRome dataset and validation dataset (S1 and S2 Tables). The discrepancy values are much lower on the LRRPredictor ground truth dataset compared to the NLRome dataset, implying that our technique makes fewer mistakes than LRRPredictor does on new data. Fig 7 demonstrates how the discrepancy is able to catch duplicate motif predictions made by LRRPredictor. These results demonstrate not only the winding number’s ability to accurately model the LRR coil, but also its generalizability to non-NLR LRR’s derived from species other than *A. thaliana*.

### Structural anomaly detection by sliding window *L*^2^ distance from Laplacian eigenvector winding number to line

**Fig 8:**
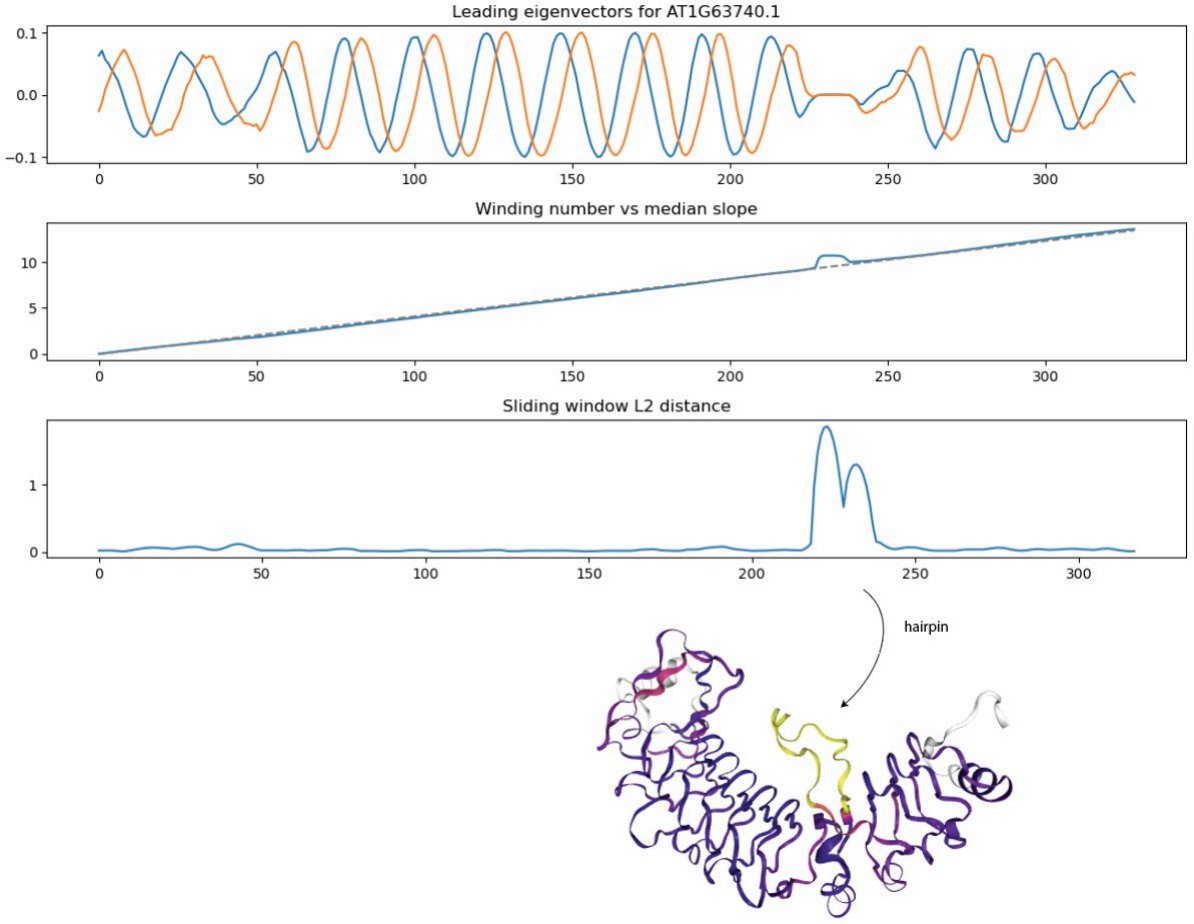
Sliding window *L*^2^ distance (SWL2D) from winding number to median secant line detects small hairpins/insertions in LRR coil domain. Structure at bottom is colored according to SWL2D where yellow values are higher.

Many LRR coils have hairpin loops and other structural anomalies which deviate from coiling. In these anomalous regions, the leading eigenvectors deviate from their usual periodic behavior. Applying the winding number formula (Eq 6) above to the pair of leading graph Laplacian eigenvectors leads to a cumulative winding number within the LRR domain which is better able to discern small hairpins compared to the previous winding number computation based on normal bundle projection. As shown in Fig 8 below, we detect a small hairpin as a spike in *L*^2^ distance between the winding number and its median slope.

## Discussion

The emergence of AlphaFold 2 has catalyzed a paradigm shift in protein structure prediction, facilitating access to genome-wide high-quality structural models. Traditional sequence homology-based domain annotation techniques, like LRRPredictor, often face challenges with LRRs, especially in proteins with high sequence divergence. While evolutionary divergence might veil the sequence homology of LRR units, their core structural topology, characterized by 20–30 amino acid stretches typically involved in protein-protein interactions, often remains conserved, acting as a distinct structural signature.

This study uses AlphaFold 2 to generate a 3D space curve from a protein sequence, which subsequently is projected into the 2D plane by identifying a series of “slinky” cross-sections. Through computing the cumulative winding number on the resultant 2D curve and employing piecewise linear regression, the linearly sloped region, identified as the LRR domain, is discerned. Our method pivots on the application of geometric data analysis to illuminate structural motifs that remain elusive to sequence analysis alone.

The use of geometric and topological concepts in our method aligns with previous studies that have explored Topological Data Analysis (TDA) in protein structure and dynamics [21, 22]. For instance, SINATRA Pro has been used to identify biophysical signatures in protein dynamics by detecting topological differences between protein structures [21]. Similarly, TopologyNet integrates TDA with deep learning for biomolecular property predictions [22]. Our approach builds on these foundational ideas by leveraging large-scale AI/ML-derived databases like AlphaFoldDB, showcasing the potential of combining AI-based structural predictions with geometric and topological analyses for advanced domain annotation. The amalgamation of advanced protein structure prediction technologies and mathematical models, as demonstrated in our approach, underscores the potential for widening our understanding of protein function across varied biological systems.

Our method yields several kinds of precise results: (*a*) it identifies the start and end sites of the LRR domain with greater accuracy than HMM-based methods, (*b*) it annotates repeat units more reliably than the existing LRRPredictor, (*c*) it identifies misannotations by other annotation/prediction tools, and (*d*) it reveals structural anomalies within the LRR domain that deviate from conventional coiling behaviors. These findings not only underscore the utility of our approach but also present a robust framework for delving into the intricate structural patterns intrinsic to LRR domains.

While we benchmarked our work on LRR domains in NLR proteins, the intrinsic methodology has the capacity for broader applications, likely extending to other linear solenoid protein domains like armadillo (ARM), tetratricopeptide (TPR), and ankyrin (ANK) repeats, all of which feature distinctive repeat sequences and structural configurations. However, the method is unlikely to work well on circular solenoid domains such as beta propellers (e.g. WD40) because, unlike linear solenoids, those structures do not consistently wind around around a core curve.

Our method does come with limitations. For instance, while it can detect non-coiling structural anomalies within the LRR domain, the origin, authenticity, and potential functionality of these regions remain ambiguous. Moreover, our structure-based annotation method, albeit effective for domains with a straightforward geometric description like LRRs, might not be universally applicable to other protein domains without developing a new geometric model tailored to them. This underscores a potential limitation when juxtaposing sequence-based versus structure-based domain annotation, highlighting a future avenue warranting exploration: developing geometric models for other protein domains.

## Supporting information

S1 Table Discrepancy values for *A. thaliana* NLRome dataset. (CSV)

S2 Table Discrepancy values for LRRPredictor training dataset. (CSV)
